# The Effect of Infection Control Nurses on the Occurrence of *Pseudomonas aeruginosa* Healthcare-Acquired Infection and Multidrug-Resistant Strains in Critically-Ill Children

**DOI:** 10.1371/journal.pone.0143692

**Published:** 2015-12-02

**Authors:** Wei Xu, Linxi He, Chunfeng Liu, Jian Rong, Yongyan Shi, Wenliang Song, Tao Zhang, Lijie Wang

**Affiliations:** Department of Pediatrics, Shengjing Hospital of China Medical University, Shenyang, Liaoning, China; University of North Dakota, UNITED STATES

## Abstract

**Background:**

Healthcare-acquired *Pseudomonas aeruginosa* (*P*. *aeruginosa*) infections in the Pediatric Intensive Care Unit (PICU), which have a high incidence, increase treatment costs and mortality, and seriously threaten the safety of critically ill children. It is essential to seek convenient and effective methods to control and prevent healthcare-acquired infections (HAIs). This research was conducted to study the effect of infection control nurses on the occurrence of *P*. *aeruginosa* HAIs and multi-drug resistance (MDR) strains in PICU.

**Methods:**

The clinical data was divided into two groups, with the age ranging from 1 month to 14 years. One group of the critically ill patients(N = 3,722) was admitted to PICU from 2007 to 2010, without the management of infection control nurses. The other group of the critically ill patients (N = 3,943) was admitted to PICU from 2011 to 2013, with the management of infection control nurses. Compare the mortality, morbidity and the incidence of acquired *P*. *aeruginosa* infections to evaluate the effect of infection control nurses.

**Results:**

After implementation of the post of infection control nurses, the patient's overall mortality fell from 4.81% to 3.73%. Among the patients with endotracheal intubation more than 48 hours, the incidence of endotracheal intubation-related pneumonia decreased from 44.6% to 34.32%. The mortality of patients with endotracheal intubation decreased from 16.96% to 10.17%, and the morbidity of HAIs with *P*. *aeruginosa* decreased from 1.89% to 1.07%. The mutual different rate (MDR) dropped from 67.95% to 44.23%. There were remarkable differences in these rates between the two groups (p<0.05).

**Conclusion:**

Implementing the post of infection control nurses is associated with effectively reducing the HAI rate, especially the incidence and morbidity of *P*. *aeruginosa* HAIs, reducing PICU mortality, improving *P*. *aeruginosa* drug resistance.

## Introduction

*Pseudomonas aeruginosa* is a Gram-negative pathogen, which is most commonly isolated in patients with nosocomial or healthcare-acquired infections (HAIs) especially pneumonia patients. Among pediatric patients, especially, this organism is prevalent in pediatric intensive care units (PICU), and its incidence as a HAI lung pathogen has doubled over the last three decades [1,2]. *P*. *aeruginosa* is intrinsically resistant to several antimicrobial agents, and it can acquire resistance to many others. In recent years the frequency of multidrug-resistant (MDR) strains of *P*. *aeruginosa* is increasing, especially in HAI and PICU-acquired infections [3–5], and these infections increase mortality, morbidity, and hospital costs. The mortality of children with *P*. *aeruginosa* infection ranges from 20% to 50% [6,7] in Chinese reports and 33% to 61% [8,9] in reports on other populations.

Among all pathogens for HAIs in PICU, *P*. *aeruginosa* has shown a rising trend in recent years and has become the primary pathogen in most regions. With the increasing severity of HAIs, *P*. *aeruginosa* drug resistance is also becoming more serious. The proportion of MDR and pan-drug resistance (PDR) strains continues to increase in some regions [10–12]. It has been shown that endotracheal intubation, central venous catheterization, various drainage tubes, and surgical/trauma incisions are primarily routes to acquire infections [13]. Aquired *P*. *aeruginosa* infection, of the majority of patients, is transmitted via the hands of medical workers or related devices. Especially in teaching hospitals like ours, interns, physicians on rotation, nurses, and visiting family members can be a variety of infective routes, making it more difficult to control *P*. *aeruginosa* HAIs. The World Health Organization (WHO), the Centers for Disease Control and Prevention (CDC) [14,15], and other organizations unceasingly emphasized the importance of controlling HAIs and highlighted appropriate methods and necessity of hand washing and timely hand disinfection. Unfortunately, numerous studies have shown that adherence to hand hygiene recommendations remains low and that improvement efforts always lack sustainability. The current worldwide focus on improving the cost-effectiveness of healthcare along with reports of successful HAI control programs, which has prompted hospital administrators, infection control teams, and researchers, and try to understand their own local infection control situations in order to improve healthcare programs [16]. Based on this reason, our hospital initiated a special infection control group in PICU in 2011, in which the most important role was the infection control nurses. As the specific infection control executors, they play an important role in the control and management of HAIs in the PICU. The main task of the nurses in this role is to educate and train new department staff and visiting families to control infection and to supervise the method and frequency of hand hygiene of relevant medical personnel in a real-time manner. They also supervise whether doctors and nurses strictly comply with the corresponding protocols in various procedures. The question is whether this post has brought some changes to the rates of *P*. *aeruginosa* HAIs. Also, is there any improvement in the prevalence of drug resistance of *P*. *aeruginosa* strains? The aim of this study was to evaluate the effect of infection control nurses on the occurrence of *P*. *aeruginosa* HAIs and MDR strains in critically-ill children. As is known, this paper is the first clinical study to specifically analyze and evaluate the role of the infection control nurses in controlling and preventing HAIs in the PICU, especially *P*. *aeruginosa* infection. It is expected that the infection control nurses can play a positive role in the development and implementation of PICU infection control policies and measures through preventing *P*. *aeruginosa* HAIs.

## Materials and Methods

The Medicine Ethics Committee of Shengjing Hospital of the China Medical University approved this study (2010PS48K). Because it was a retrospective study, which was exempt from patient, parent or guardian informed consent. Clinical data was collected in a manner that can maintain patients' privacy. Patients' records or information was anonymized and de-identified prior to analysis. Inclusion criteria: 1. Pediatric patients aged 1 month to 14 years hospitalized in the PICU of Shengjing Hospital of China Medical University from January 1, 2007 to December 31, 2013. 2. Based on the patient’s condition, blood culture, sputum culture, and catheter tip culture at the time of replacement of endotracheal tube or at extubation in patients with mechanical ventilation or endotracheal intubation, as well as catheter tip culture at the time of removal of central venous catheter, and secretion (e.g., localized pus, pleural effusion, cerebrospinal fluid, urine) cultures were performed. Confirmed diagnosis of *P*. *aeruginosa* infection: 2 times of positive cultures of the corresponding specimens except endotracheal tube tips which were used to help assess the colonization, with an interval of 3 to 5 days between each culture, with the same strains; or a single positive blood, cerebrospinal fluid, drainage fluid, or bronchoalveolar lavage fluid culture, with clinical signs and symptoms associated with *P*. *aeruginosa* infection. All positive *P*. *aeruginosa* cultures were collected. According to the definition of community-acquired infection and HAI, it was determined whether the strains were HAIs.

Setting and duties of infection control nurses: As of January 1, 2011, a special infection control post was established, as 1 nurse per 10 beds, with coverage provided during all shifts. And all these nurses were also in charge of primary nursing work. How do *P*. *aeruginosa* HAI rates changed after the intervention of the infection control nurses? They try to improve hand hygiene and hand washing compliance and to ensure the standard of disposable supplies and device disinfection, which timely identify patients with *P*. *aeruginosa* infection, and maximally reduce the transmission channels of *P*. *aeruginosa* infection.

Job description: 1. Train and supervise doctors, nursing staff, cleaning staff, and other personnel to correctly implement various disinfection measures and performance of disinfection and isolation techniques. 2. Supervise and remind of hand washing and hand disinfection of medical staff. 3. Immediately mark on bed signs regarding infection risk and isolate confirmed or suspected patients with *P*. *aeruginosa* HAI, and limit all routes of transmission. 4. Increase specimen detection rate before administration of empiric medication. 5. Ensure no repeated use of disposable medical devices and apparatus and encourage using disposable materials instead of similar devices sterilizing repeatedly. 6. Implement separate use of hand disinfectants and stethoscopes, percussion hammers, pupil pens, and other diagnostic and treatment instruments for each patient.

Community-acquired, healthcare-associated, and hospital-acquired infections were defined using the criteria of the Centers for Diseases Control and Prevention (CDC) [17].

The clinical criteria for the diagnosis of VAP: Patients who are mechanically ventilated for equalor greater than 48 h must have two or more abnormal chest radiographs with at least one of the following symptoms: new or progressive and persistent infiltrate, consolidation, cavitation, and/or pneumatoceles (infants age≤1 year). However, in patients without underlying pulmonary or cardiac disease (respiratory distress syndrome, bronchopulmonary dysplasia, pulmonary edema, or chronic obstructive pulmonary disease), one definitive chest radiograph is acceptable. In addition to abnormal chest radiographs, a patient must have at least one of the following symptoms: fever (>38°C) with no other exact cause, leukopenia (<4,000 white blood cells [WBC]/mm^3^) or leukocytosis (≥12,000 WBC/mm^3^), and at least two of the following criteria: new onset of purulent sputum, change in character of sputum, increased respiratory secretion, or increased suctioning requirements; new onset of or aggravating cough, dyspnea, or tachypnea; rales or bronchial breath sounds; and worsening gas exchange (e.g., O_2_ desaturations [e.g., PaO_2_/FiO_2_ levels ≤240], increased oxygen requirements, or increased ventilation demand).

Strain identification and susceptibility testing: API system and VlTEK 2-COMPACT system (BioMérieux, France) were employed for identification of *P*. *aeruginosa* strains. Both disk diffusion (Kirby-Bauer method) and broth microdilution methods were used in vitro susceptibility testing. Testing was in strict accordance with the method recommended by the Clinical and Laboratory Standard Institute (CLSI) [18]. The quality control strain was *P*. *aeruginosa* ATCC27853. The minimum inhibitory concentrations (MIC) of 16 antibiotics were analyzed, and the susceptibility testing results were evaluated according to the National Committee for Clinical Laboratory Standards (NCCLS) [19] and reported sensitive (S), intermediate (I), or resistant (R). Both intermediate and resistant strains were classified as resistant strains.

Antimicrobial drugs and reagents: The 16 antibiotics were used for drugs resistance test. The susceptibility disk was the product of British Oxoid Limited. The susceptibility culture medium was Müller-Hinton (M-H) agar produced by BioMerieux. A Walkaway96 series susceptibility plate from US Siemens was employed for broth microdilution method.

Criteria for MDR and PDR [20]: MDR was defined as a strain non-susceptible to ≥1 agent in ≥3 antipseudomonal antimicrobial categories including penicillins, cephalosporins, aminoglycosides, fluoroquinolones and/or carbapenems or other antibiotics at the same time. PDR was defined as non-suscetibility to all agents in all antimicrobial categories except polymyxin.

Grouping and study program: According to the time point of setting infection control nurses, the patients in the study were divided into two groups—inpatients from January 1, 2007 to December 31, 2010 in Group A and inpatients from January 1, 2011 to December 31, 2013 in Group B. Patients were also grouped according to the location where *P*. *aeruginosa* infection was acquired—community-acquired infection and HAI groups. General information was analyzed retrospectively in both two Groups, and the relationships between strain specimen source, disease distribution, invasive operation of endotracheal intubation and central venous catheterization, trauma or surgery and other related risk factors and hospital-acquired *P*. *aeruginosa* infection in different groups were compared and analyzed. Besides, the susceptibility testing of different strains was summarized and analyzed. That the resistance rates to different drugs and the effects of the infection control nurses on drug resistance were compared and analyzed. The modified child critical illness scores [21] was used to evaluate the illness severity of all children admitted in PICU in this period.

Statistical analysis: Data analysis was conducted using the SPSS 13.0 software. Discrete variables were expressed as counts (percentages), and continuous variables are expressed as means ± standard deviation (SD). Differences in the demographic and clinical characteristics of the patient groups were assessed using a chi-square test for categorical variables. As data showed a normal distribution, one-way analysis of variance (ANOVA) was adopted to analyze inter-subgroup differences. For data that did not show a normal distribution, a Kruskal-Wallis test was used to analyze inter-subgroup differences. P≤0.05 was considered statistically significant.

## Results

### 1. Demographics and clinical manifestations of all patients and burdens of endotracheal intubation (Tables 1 and 2)

**Table 1:** General characteristics of all patients admitted between 2007 and 2013.

	A(2007–2010)	B(2011–2013)	P value
Patient cases	3722	3943	
Gender (male, n, %)	2204 (59.22%)	2382 (60.41%)	0.296655
Age (months, median)	18	16	0.74326
Child critical illness score^a^	82.46±12.78	77.76±14.63	0.01935
Number of patients undergoing endotracheal intubation (n, %)	572 (15.37%)	708 (17.96%)	0.002653
Duration of endotracheal intubation (days, median)	9 (3–99)	11 (3–112)	0.4673
Endotracheal intubation associated pneumonia (n, %)	255/572 (44.6%)	243/708 (34.32%)	0.000229
Central venous catheterization (n, %)	95 (2.55%)	129 (3.27%)	0.071762
Femoral vein (n, %)	64 (1.72%)	78 (1.98%)	
Jugular vein (n, %)	13 (0.35%)	2 1(0.53%)	
Subclavian vein (n, %)	3 (0.08%)	7 (0.18%)	
PICC (via the cubital vein) (n, %)	15 (0.4%)	23 (0.58%)	
Trauma and after invasive surgery (n, %)	769 (20.66%)	946 (23.99%)	0.000521
Average length of stay (days, median)	11 (0–114)	12 (0–146)	0.7473
Cost per patient (US Dollar)	4098.36±2642.32	4293.52±3011.45	0.3761
Total deaths (n, %)	179 (4.81%)	147 (3.73%)	0.022160
Deaths from endotracheal intubation for more than 48 h^b^ (n, %)	97/572 (16.96%)	72/708 (10.17%)	0.000494
Deaths from central venous catheterization for more than 24 h^b^ (n, %)	12/95 (12.63%)	18/129 (13.95%)	0.929393

^a^Critical illness score refers to scores of patients within 24 hours after admission according to the Modified Child Critical Illness Score.

^b^Given that the vast majority of endotracheal intubation-associated infections occurred in patients intubated for more than 48 hours and central venous catheterization-associated infections did happen for more than 24 hours after setting.

**Table 2:** Main burdens of patients undergoing endotracheal intubation with or without healthcare-associated pneumonia.

	Endotracheal intubation and non-healthcare-acquired pneumonia (n = 781)	Endotracheal intubation-associated non-*P*. *aeruginosa* pneumonia (n = 408)	Endotracheal intubation-associated *P*. *aeruginosa* pneumonia (n = 91)	P value
Duration of endotracheal intubation (days, median)^a^	7 (3–22)	13 (5–102)	16 (6–112)	0.001455
Average length of stay (days, median, range)^b^	17 (10–35)	24 (12–114)	29 (14–146)	0.003742
Per patient hospital costs^a^ (USD)	15,5479	26,0982	29,7654	0.000184
Mortality (n, %)^b^	73 (9.35)	77 (18.87)	29 (31.87)	0.00000

^a^There was no significant difference between *P*. *aeruginosa* and non- *P*. *aeruginosa* groups.

^b^There was significant difference between *P*. *aeruginosa* and non- *P*. *aeruginosa* groups (p<0.05). Not all patients died due to secondary *P*. *aeruginosa* pneumonia, but *P*. *aeruginosa* pneumonia increased the risk of death in pediatric patients.

The PICU of Shengjing Hospital of China Medical University treated a total of 7,665 patients from 2007 to 2013, including 3,722 patients (Group A) before and 3,943 (Group B) after creation of the post of the infection control nurses. The modified child critical illness scores in Group B were lower than that in Group A, suggesting that the children in Group B were more critically ill when admitted in. A total of 1,647 patients underwent endotracheal intubation in order to provide mechanical ventilation support or to relieve upper airway obstruction. In Group A, 729 patients were intubated, with 15.37% (572/3722) intubated for more than 48 hours. In Group B, 918 patients were intubated, 17.96% (708/3943) intubated for more than 48 hours. The difference between the two groups was significant (p<0.05). In the 572 patients intubated in Group A, 255 (44.6%) patients developed an endotracheal intubation-associated pneumonia, significantly higher than the 243 (34.32%) in Group B (p<0.05). A total of 224 patients were applied to central venous catheterization more than 24 hours, among whom 132 cases were treated for Continuous Blood Purification; 47 cases were used for monitoring of circulatory function; 29 cases were operated for parenteral nutrition, and 16 cases were for other reasons. The placement of central venous catheterization was mainly in the femoral vein, internal jugular vein, subclavian vein, and as peripherally-inserted central catheter (PICC) via the cubital vein. A total of 1,715 patients had trauma or invasive surgery, among whom more than 20%. trauma patients accounted for 56.91%(n = 976) of all patients, mainly on account of traffic injuries, injuries due to falling from heights, or burns. While the remaining 739 patients had postoperative critical illnesses, mainly including cardiac surgeries, neurosurgery, abdominal and thoracic surgeries. There were 769 (20.66%) trauma and postoperative patients in Group A and 946 (23.99%) in Group B. The difference between the two groups was significant (p<0.05). A total of 340/7665 (4.44%) patients died, including 179 (4.81%) deaths in Group A and 147 (3.73%) deaths in Group B. The difference in mortality between the two groups was significant (p<0.05). Among patients undergoing endotracheal intubation more than 48 hours, 169/1280 (13.2%) patients died, including 97/572 (16.96%) deaths in Group A and 72/708 (10.17%) deaths in Group B. The difference in mortality between the two groups was significant (p<0.05). Dividing both group A and B into two subgroups based on ages as 1month-3 years old and 4 years old -14 years old, we compared the data of demographics of patients in group A and B within same age group. The trend of the results was similar with the results reported in the tables.

Among the 7,665 patients, 1,280 patients exceeded the duration of endotracheal intubation for 48 hours. The 1,280 patients were then divided into three groups, according to whether the patients developed pneumonia secondary to endotracheal intubation and whether the pathogen was *P*. *aeruginosa*. In the patients with confirmed infections, 91 (7.1%) had identified *P*. *aeruginosa* and 408 (31.88%) had infections of other types of bacteria, including *Acinetobacter baumanni*, of 73 cases; *Klebseilla pneumonia of* 69 cases; *Escherichia coli of* 53 cases; *Staphylococcus aureus of* 47 cases; *Enterobacter cloacae of* 29 cases; *Enterococcus faecium of* 26 cases; *Streptococcus pneumonia of* 26 cases; *Pseudomonas cepacia* of 23 cases; coagulase negative staphylococcus of 15 cases; *Enterococcus faecalis of* 12 cases; *Stenotrophomonas maltophilia of* 9 cases; and other microorganism of 20 cases. It was found that the average duration of endotracheal intubation and average length of stay in the endotracheal intubation-associated *P*. *aeruginosa* pneumonia group and non-*P*. *aeruginosa* group was significantly longer than that in the group without endotracheal intubation-associated pneumonia. The average hospital costs and mortality both significantly increased in the two groups of patients with secondary pneumonia (p<0.05).

### 2. Characteristics of 130 patients with P. aeruginosa infections (Tables 3–6)

**Table 3:** Characteristis of patients with P. aeruginosa infection.

Characteristics	A(2007–2010)	B(2011–2013)	P value
Hospitalized patients	3722	3943	
P. aeruginosa infection cases (n, %)	78 (2.10%)	52 (1.31%)	0.010476
Community infections (n, %)	8 (0.21%)	10 (0.25%)	0.909589
Healthcare-associated infections (n, %)	70 (1.89%)	42 (1.07%)	0.003993
Total number of culture-positive specimensa (n, %) ^a^	147 (3.95%)	113 (2.87%)	0.010581
Total number of positive specimens from infection patients (n, %)	112 (3.01%)	85 (2.16%)	0.022159
Total number of positive specimens from community-acquired infections (n, %)	21 (0.56%)	26 (0.66%)	0.698649
Total number of positive specimens from healthcare-acquired infections (n, %)	91 (2.45%)	59 (1.50%)	0.021675
Mortality rate for P. aeruginosa infection (n, %)	30/78(38.46%)	18/52 (34.15)	0.795112
Mortality rate for healthcare-associated P. aeruginosa infection (n, %)	26/70 (37.14%)	13/42 (30.95)	0.364346

^a^Including colonization strains. Colonization strains were primarily specimens that had one positive sputum or catheter culture, and patient’s clinical presentation was not typical of *P*. *aeruginosa* infections and were, therefore, not treated as culture-positive strains.

**Table 4:** Effect of the infection control nurse on the sites of community-acquired and healthcare-acquired P. aeruginosa infections.

	Community-acquired infections (n = 18)		p value	Healthcare-acquired infections (n = 112)		P value
	A(2007–2010, n = 8)	B(2011–2013, n = 10)		A(2007–2010, n = 70)	B(2011–2013, n = 42)	
Lower respiratory tract (n, %)	1 (12.5%)	2 (20%)	0.832004	58 (82.86%)	33 (78.57%)	0.754632
Gastrointestinal tract (n, %)	6 (75%)	7 (70%)	0.768625	2 (2.86%)	3 (7.14%)	0.554731
Central venous catheter (n, %)				6 (8.57%)	4 (9.52%)	0.864132
Other^a^ (n, %)	1 (12.5%)	1 (10%)	0.557225	4 (5.71%)	2 (4.76%)	0.828440

^a^Other infections included surgical incisions, trauma wounds, burns, bone/joint, and other sites.

**Table 5:** The distribution of community-acquired and healthcare-acquired P. aeruginosa infection positive specimens.

	Community-acquired infection positive specimen (n = 47)		p value	Healthcare-acquired infection positive specimen (n = 150)		P value
	A(2007–2010, n = 21)	B(2011–2013, n = 26)		A(2007–2010, n = 91)	B(2011–2013, n = 59)	
Blood (n, %)	8 (38.1%)	9 (34.6%)	0.953380	8 (8.79%)	6 (10.17%)	0.996944
Bronchoalveolar lavage (n, %)	1 (4.76%)	3 (11.54%)	0.762644	10 (10.99%)	8 (13.56%)	0.828964
Tracheal cannula (n, %)				23 (25.27%)	10 (16.95%)	0.316985
Sputum (n, %)	0 (%)	1 (3.85%)		35 (38.46%)	28 (47.46%)	0.356973
Pus^a^ (n, %)	12 (57.14%)	13 (50%)	0.846245	9 (11.54%)	3 (5.08%)	0.308701
Catheter (n, %)				6 (6.59%)	4 (6.8%)	0.771535
Total	21 (0.56%)	26 (0.66%)		91 (2.44%)	59 (1.5%)	

^a^Pus includes purulent secretions, cerebrospinal fluid, peritoneal purulent secretions, and osteomyelitis drainage fluid of purulent necrotic tissue of skin soft tissue caused by bloodstream infection and other wounds after *P*. *aeruginosa* infection; this group primarily compares whether there was any difference in the distribution of sources of positive specimens between the two groups.

**Table 6:** Effect of infection control nurse on the incidence of acquired P. aeruginosa under various conditions.

	A(2007–2010, n = 3722)	B(2011–2013, n = 3943)	P value
Endotracheal intubation-associated infection (n, %)	58/572 (10.14%)	33/708 (4.66%)	0.000231
Venous catheter-associated bloodstream infection (n, %)	6/95 (6.32%)	4/129 (3.1%)	0.381774
Infection after trauma and surgery (n, %)	4/769 (0.52%)	2/946 (0.21%)	0.505560
Other infections	2	3	
Total	70	42	

During the 7-year study period, a total of 130/7665 (1.7%) patients were treated and diagnosed with *P*. *aeruginosa* infections. The proportion of patients in Group A (n = 78, 2.10%) was higher than that in Group B (n = 52, 1.31%), and the difference between the two groups was significant (p<0.05). There were 112/130 (86.15%) of all patients with HAIs; The proportion of patients in Group A (n = 70, 1.89%) was higher than that in Group B (n = 42, 1.07%), and the difference between the two groups was significant (p<0.05). There were a total of 260/7665 (3.39%) positive specimens; and the proportion of positive specimens in Group A (n = 147, 3.95%) was higher than that in Group B (n = 113, 2.87%). The difference between the two groups was significant (p<0.05). There were 197/7665 (2.57%) positive specimens from patients with infections. The proportion of positive specimens in Group A (n = 112, 3.01%) was higher than that in Group B (n = 85, 2.16%), and the difference between the two groups was significant (p<0.05). Patients with HAIs had 150 positive specimens; the proportion of positive specimens in Group A (n = 191, 2.45%) was higher than that in Group B (n = 59, 1.50%). The difference between the two groups was significant (p<0.05).

*P*. *aeruginosa* can cause infections at various sites and spread to a systemic infection. According to the initial patient clinical symptoms and signs and corresponding laboratory tests, the initial sites of acquired *P*. *aeruginosa* infection were primarily lower respiratory tract (pneumonia), gastrointestinal tract (diarrhea, sepsis, shock), central venous catheterization, local infection after surgery or trauma, and infection of drainage tubes. In the 18 patients with community-acquired infections, 3 cases had middle and lower respiratory tract infections, manifesting as severe pneumonia; 1 case was associated with lung abscess,; 1 case was complicated by pyothorax, and 1 case manifested as hypoxemia. The gastrointestinal tract was the primary site of infection in 13/18 (72.22%) patients, which was the main site for community-acquired severe *P*. *aeruginosa* infection. In addition to vomiting, diarrhea, and dehydration, all 13 patients had severe sepsis and all of them were admitted to PICU. This included 10 patients with septic shock; 2 cases with purulent meningitis, and 1 case with acute peritonitis. There were 8 patients with soft tissue cellulitis associated with skin necrosis. Two patients had other infections. One patient had fever associated with joint swelling and tenderness and was diagnosed with *P*. *aeruginosa of* right femoral osteomyelitis. Another patient had local skin abscesses duo to mosquito bites. Lower respiratory tract infections accounted for the majority (91/112, 81.25%) of the 112 patients HAIs, manifesting primarily as severe pneumonia. Central venous catheterization-associated *P*. *aeruginosa* bloodstream infections occurred in 13/112 (11.61%) patients. Five patients had a secondary infection of the gastrointestinal tract and 2 patients with septic shock and died of intestinal necrosis. Other *P*. *aeruginosa* infections occurred in 6/112 (5.32%) patients, including 4 in Group A (1 surgical incision infection, 1 skin soft tissue infection after perineal laceration, 1 burn infection, 1 infection after cystostomy) and 2 patients in Group B (1 pelvic infection after pelvic fracture and 1 intracranial infection from a ventricular drain placed after medulloblastoma surgery). There were 70 patients with *P*. *aeruginosa* HAIs in Group A and 42 patients in Group B, and the difference in the primary site of infection between the two groups was not significant (p>0.05).

Forty-seven *P*. *aeruginosa* positive specimens were obtained from patients with community-acquired infections; the largest number of positive specimens were from pus (n = 25, 53.19%). Patients with HAIs had 150 *P*. *aeruginosa* positive specimens; there were 91 HAI positive specimens in Group A and 59 positive specimens in Group B. Although the rates of sputum positive specimens in Group A were much higher than those in Group B, there was no significant difference in the distribution of the positive rate of various specimens between the two groups (p>0.05). A total of 112 patients were diagnosed with *P*. *aeruginosa* HAIs. Prior to the infection control nurses intervention, 572 patients in Group A underwent endotracheal intubation more than 48 hours; 58 patients (10.14%) were diagnosed with healthcare-acquired *P*. *aeruginosa* pneumonia. In Group B, 708 patients were intubated more than 48 hour; 33 patients (4.66%) were diagnosed with healthcare-acquired *P*. *aeruginosa* pneumonia. The incidence of endotracheal intubation-associated healthcare-acquired *P*. *aeruginosa* pneumonia in Group A was far higher than that in Group B, and the difference between the two groups was significant (p<0.05). The incidence of acquired *P*. *aeruginosa* bloodstream infection in patients receiving central venous catheterization and the incidence of acquired *P*. *aeruginosa* infection in patients with trauma and invasive surgery were significantly lower than that in patients receiving endotracheal intubation, and the difference between the two groups was not significant (p>0.05).

### 3. Antibiotic Resistance of P. aeruginosa Isolated from Patients (Tables 7 and 8)

**Table 7:** Analysis and comparison of drug resistance of 130 strains of P. aeruginosa in patients with infection.

	A(2007–2010, n = 78)					B(2011–2013, n = 52)				^a^P value
	2007 (n = 16)	2008 (n = 21)	2009 (n = 19)	2010 (n = 22)	Total	2011 (n = 19)	2012 (n = 15)	2013 (n = 18)	Total	
Polymyxin B	0	0	0	1 (4.17)	1 (1.28)	3 (15.79)	0	0	3 (5.77)	0.350807
Ciprofloxacin	3 (18.75)	5 (23.81)	2 (10.53)	2 (9.09)	12 (15.38)	2 (10.52)	2 (13.33)	2 (11.11)	6 (11.54)	0.716722
Cefoperazone/sulbactam	3 (18.75)	12 (57.14)	3 (15.79)	7 (31.82)	25 (32.05)	5 (26.31)	4 (26.67)	8 (44.44)	17 (32.69)	0.908566
Meropenem	5 (31.25)	13 (61.90)	5 (26.32)	7 (31.82)	30 (38.46)	2 (10.52)	4 (26.67)	4 (22.22)	10 (19.23)	0.032890
Ceftazidime	4 (25.0)	16 (76.19)	6 (31.58)	9 (40.91)	35 (44.87)	7 (36.84)	7 (46.67)	4 (22.22)	18 (34.61)	0.325281
Cefoperazone	6 (37.5)	16 (76.19)	5 (26.32)	9 (40.91)	36 (46.15)	6 (31.58)	6 (40)	7 (38.89)	19 (36.54)	0.364974
Aztreonam	6 (37.5)	17 (80.95)	5 (26.32)	12 (54.55)	40 (51.28)	8 (42.11)	5 (33.33)	6 (33.33)	19 (36.54)	0.140393
Piperacillin/Tazobactam	13 (81.25)	17 (80.95)	6 (31.58)	7 (31.82)	43 (55.13)	9 (36.84)	8 (53.33)	5 (27.78)	20 (38.46)	0.092246
Imipenem and Cilastatin Sodium	7 (43.75)	16 (76.19)	8 (42.11)	12 (54.55)	43 (55.13)	7 (36.84)	5 (33.33)	4 (22.22)	16 (30.77)	0.010677
Amikacin	14 (87.5)	18 (85.71)	8 (42.11)	7 (31.82)	47 (60.26)	11 (57.89)	8 (53.33)	7 (38.89)	26 (50)	0.329976
Cefepime	15 (93.75)	18 (85.71)	9 (47.37)	8 (36.36)	50 (64.1)	9 (47.37)	9 (60)	8 (44.44)	26 (50)	0.156524
Ceftriaxone	15 (93.75)	21 (100)	17 (89.47)	22 (100)	75 (96.15)	15 (78.95)	13 (86.67)	16 (88.89)	44 (84.62)	0.046136
Cefotaxime	16 (100)	21 (100)	19 (100)	22 (100)	78 (100)	19 (100)	15 (100)	18 (100)	52 (100)	
Cefazolin	16 (100)	21 (100)	19 (100)	22 (100)	78 (100)	19 (100)	15 (100)	18 (100)	52 (100)	
Cefuroxime	16 (100)	21 (100)	19 (100)	22 (100)	78 (100)	19 (100)	15 (100)	18 (100)	5 (100)	
Ampicillin	16 (100)	21 (100)	19 (100)	22 (100)	78 (100)	19 (100)	14 (93.33)	17 (94.44)	50 (96.13)	

^a^P value for the comparison of overall drug resistance of each antibiotic before and after infection control nurses interventions.

**Table 8:** Changes in multi-drug and pan-drug resistance of *P*. *aeruginosa* strains isolated from 130 patients.

		MDR (n, %)	PDR (n, %)
A	2007 (n = 16)	14 (87.5)	0
	2008 (n = 21)	16 (76.19)	4 (19.04)
	2009 (n = 19)	11 (57.89)	3 (15.79)
	2010 (n = 22)	12 (54.55)	2 (9.09)
Total (n = 78)		53 (67.95)	9 (11.54)
B	2011 (n = 19)	7 (36.84)	1 (5.26)
	2012 (n = 15)	8 (53.33)	2 (13.33)
	2013 (n = 18)	8 (44.44)	1 (5.56)
Total (n = 52)		23 (44.23)	4 (7.69)
P		0.012185	0.676142

Susceptibility tests with 16 antibiotics for the *P*. *aeruginosa* strains were isolated from 130 patients with community-acquired infections and HAIs all showed high resistance rates. However, after the intervention of infection control nurses, the rate of drug resistance improved. The resistance rate to 11 of the antibiotics decreased; resistance rates to meropenem, piperacillin/tazobactam, imipenem/cilastatin sodium, and ceftriaxone significantly decreased, and the difference between the two groups was significant (p<0.05).

With the decrease in *P*. *aeruginosa* infection rate and resistance rates of the strains to a variety of antibiotics, the incidence of MDR strains also changed. After the creation of the post of the infection control nurses, the MDR rate decreased from 67.95% to 44.23%, and there were significant differences between the two groups (p<0.05).

## Discussion

In our study, consistent with many other studies [12,22], community-acquired *P*. *aeruginosa* infections and *P*. *aeruginosa* HAIs both had a high mortality. *P*. *aeruginosa* HAIs accounted for the majority of patients with *P*. *aeruginosa* infection in the PICU, at 112/130 (86.15%) in this study. Acquired infection of *P*. *aeruginosa* increased the difficulty in treating critical pediatric patients, prolonged the treatment duration and length of stay, increased the average costs during hospitalization, and carried a high mortality. An overall case fatality rate of 52% was reported in a study of *P*. *aeruginosa* bacteremia in children hospitalized at a single center in southern China [6]. As most patients are infected with *P*. *aeruginosa* during hospitalization, it is important to find an effective policy to prevent the organism from spreading. In the past decade, patients and physicians had benefited from the introduction of several methods of prevention of *P*. *aeruginosa* HAIs, targeted at decreasing secondary infections in the hospital, such as hand hygiene, controlling environmental contamination, personnel protection, special patient-care equipment, discarding disposable stuff, and use of antiseptics [23]. Despite the continuing concern of hospital managers and all attempts at improvement, many healthcare institutions were unable to achieve adequate levels of prevention, particularly in developing countries. Meanwhile, in academic institutions like our hospital, there would be many staffs, such as medical students, new interns, and non critical care clinicians, who would not familiar with the protocols and rules of HAIs prevention and had a higher risk of spreading *P*. *aeruginosa*.

From a global perspective, acknowledgment that HAIs do occur and that many of these infections are preventable is an obvious prerequisite for improvements in infection control in any country. Infection control professionals require training and experience in a complex array of infectious diseases, epidemiology, microbiology, biostatistics, informatics, healthcare management, patient-care practices, adult education, and behavioral science [24].

First of all, we analyzed and compared the severity of critical patients in the PICU and the risk factors for HAIs before and after the creation of the post of infection control nurses [25] as well as the rates of HAIs between the two groups. The critical illness scores of all patients in Group B were lower than in Group A. There were more patients with trauma and invasive surgery in Group B than in Group A, and the number and rate of patients receiving central venous catheterization in Group B were also significantly higher than in Group A. Those all suggested that patients in Group B were in more critical condition when admitted to hospital compared with those in Group A. In addition, the number of patients with endotracheal intubation for more than 48 hours in Group B was significantly higher than in Group A. However, the incidence of endotracheal intubation-associated pneumonia and central venous catheterization-associated infection in Group B was significantly decreased, and the mortality of patients intubated for more than 48 hours was also significantly lower than in Group A. The overall patient mortality in Group B was significantly lower than that in Group A. These data suggest that the interventions of the infection control nurses, for the control of HAIs yield prominent effects in critical pediatric patients in the PICU. Long-term endotracheal intubation was the most significant risk factor for PICU-acquired pneumonia [26,27]. In this study, the mortality for patients intubated for more than 48 hours was higher than the mortality for the general patients. Among all 1,280 patients intubated for more than 48 hours, 499 developed pneumonias secondary to bacterial infections; the number of patients with *P*. *aeruginosa* pneumonia was associated with a longer duration of endotracheal intubation and prolonged hospital stay, as well as higher hospital costs and mortality. Haley and his colleagues indicated that HAIs were among the top 10 leading causes of death in the United States [16]. HAIs were an important public health problem in Brazil, with 11 million hospital admissions per year and a rate of HAIs of 5% to 10% [28].

Moreover, the diseases distribution, specimen sources, and prognosis of patients with community-acquired and healthcare-acquired *P*. *aeruginosa* were compared and analyzed. After the efforts of the infection control nurses, the *P*. *aeruginosa* infection rate, especially the *P*. *aeruginosa* HAI rate, was significantly decreased, as well as the rate of positive specimens. Especially, the rate of positive specimens of *P*. *aeruginosa* infected patients decreased significantly after setting the post of infection control nurses, so did the positive rate of specimens of patients with HAIs. On the contrary, these rates underwent no significant changes in patients with community-acquired *P*. *aeruginosa* infections. Meanwhile, to avoid the deviation produced by outbreaks of epidemic diseases in specimen sources, community-acquired and healthcare-acquired *P*. *aeruginosa* infected specimen rates were analyzed and compared for all specimens including blood, endotracheal tube tips, bronchoalveolar lavage fluid, venous catheter tips, sputum, and purulent secretions before and after the interventions of the infection control nurses and found no significant difference. Thus, the effect of epidemic diseases or disease outbreaks in different time periods could be largely ruled out, such as H1N1 infection, hand-foot and mouth disease, and measles. Healthcare-acquired *P*. *aeruginosa* infections primarily included endotracheal intubation-associated pneumonia, central venous catheterization-associated bloodstream infection, infections after trauma and invasive surgery, and other types of infections [26,27,29–31]; endotracheal intubation-associated *P*. *aeruginosa* pneumonia accounted for the majority of HAIs (91/112, 81.25%). The infection control nurses played a very important role in reducing endotracheal intubation-associated *P*. *aeruginosa* pneumonia. After the implementation of the infection control nurses, the incidence of secondary *P*. *aeruginosa* pneumonia in patients intubated for more than 48 hours decreased from 10.14% to 4.66%. These data also suggest that HAIs were mainly related to invasive procedures and treatments, and the supervision by infection control nurses effectively guaranteed hand hygiene and significantly reduced *P*. *aeruginosa* transmission and planting associated with various treatments and procedures.

Infections caused by *P*. *aeruginosa* are often difficult to treat; inappropriate antimicrobial readily selects MDR *P*. *aeruginosa* [32]. In order to optimize the initial management of children with serious *P*. *aeruginosa* infections, early recognition of the infection by clinicians and appropriate antimicrobial selection are critical. Therefore, it is necessary to understand the clinical manifestations and patterns of antimicrobial resistance with *P*. *aeruginosa*. *P*. *aeruginosa* possesses an intrinsic resistance to many antimicrobials because of the bacterium’s outer membrane barrier, the presence of multidrug efflux transporters, and endogenous antimicrobial inactivation [33]. Although anti-pseudomonal agents (e.g., carbapenems) have been discovered and developed, *P*. *aeruginosa* readily acquires resistance to individual agents via chromosomal mutations and lateral gene transfer [33]. The incidence of MDR *P*. *aeruginosa* infections is associated with increased morbidity, mortality, and cost [34]. This organism may be exposed to a wide range of concentrations of antimicrobials during treatment, so it is first necessary to learn more about the responses of *P*. *aeruginosa* to antimicrobials and to understand how the pattern of its antimicrobial resistance changes, which may be key to antimicrobial selection.

As was mentioned above, the infection control nurses significantly reduced the rate of HAI with *P*. *aeruginosa* and, consequently, the use of broad-spectrum antibiotics will be decreased, as will resistant strains of *P*. *aeruginosa* induced by antibiotic use. Through analysis of *P*. *aeruginosa* susceptibility tests before the interventions of the infection control nurses, we found that carbapenems, which had previously been widely used by clinicians, and piperacillin/tazobactam both had high resistance rates. To protect the clinical activity of these antibiotics, after the infection control nurses, the use of carbapenems and piperacillin/tazobactam was minimized for the treatment of *P*. *aeruginosa* infections. This resulted in a significant drop in the rate of resistance to these antibiotics in recent years, while the resistance rates to cefoperazone/sulbactam, ceftazidime, and other increasingly used antimicrobials have not been significantly increased. This is also the main reason why MDR strains of *P*. *aeruginosa* isolated from patients were significantly decreased after the interventions of the infection control nurses in this study. Infection with an MDR strain was also a high risk factor for increased mortality in patients with *P*. *aeruginosa* infection [34]. The resistance rate to ciprofloxacin was the lowest of all of the antimicrobials in this study, and far lower than the resistance rate to ciprofloxacin in adults (33% to 50%) [11,35,36]. This may be related to the fact that quinolones have not been approved for the treatment of infections in children. But because *P*. *aeruginosa* infections are difficult to control, we occasionally use these agents in the PICU after obtaining the consent of a parent or guardian. Due to the limitations of drug availability and lack of effectiveness for refractory *P*. *aeruginosa* infections, carbapenems are more likely to be chosen for use in the PICU, which may be the reason why the resistance rate to these drugs was up to 30% to 50% before the infection control nurses post was created, but was relatively lower in adult patients [10,36]. Relationship between bacteria MDR, mortality of patients with VAP and antibiotics treatment would be studied in the near future. Further studies are needed to determine whether broad-spectrum antibiotic treatment is cost-effect efficient in these patients.

*Pseudomonas aeruginosa* is one of the major pathogens responsible for a wide variety of severe healthcare- and community-acquired infections. Numerous vaccine candidates and several monoclonal antibodies have been developed over the past 40 years, but only a few have reached clinical trials, and none of these vaccine candidates has obtained market authorization. Therefore, adoption of various possible means to prevent and control the occurrence of *P*. *aeruginosa* HAIs, early identification of the serious infection caused by such pathogens, and selection of appropriate antimicrobials are still the main means to reduce patient morbidity/mortality and family burdens.

## Conclusion

Implementing a post of infection control nurses might be associated with effectively reducing the HAI rate, especially the rate of *P*. *aeruginosa* HAI, reducing PICU mortality, improving *P*. *aeruginosa* drug resistance, and promoting the safety of critical patients in the PICU.

## References

[1] Jones RN (2010) Microbial etiologies of hospital-acquired bacterial pneumonia and ventilator-associated bacterial pneumonia. Clin Infect Dis 51 Suppl 1: S81–87. 10.1086/653053 20597676

[2] Torres A, Ewig S, Lode H, Carlet J, European HAPwg (2009) Defining, treating and preventing hospital acquired pneumonia: European perspective. Intensive Care Med 35: 9–29. 10.1007/s00134-008-1336-9 18989656

[3] Bayani M, Siadati S, Rajabnia R, Taher AA (2013) Drug Resistance of Pseudomonas aeruginosa and Enterobacter cloacae Isolated from ICU, Babol, Northern Iran. Int J Mol Cell Med 2: 204–209. 24551814 PMC3927384

[4] Falagas ME, Sideri G, Vouloumanou EK, Papadatos JH, Kafetzis DA (2009) Intravenous colistimethate (colistin) use in critically ill children without cystic fibrosis. Pediatr Infect Dis J 28: 123–127. 10.1097/INF.0b013e31818a5dbd 19116601

[5] Folgori L, Livadiotti S, Carletti M, Bielicki J, Pontrelli G, Ciofi Degli Atti ML, et al (2014) Epidemiology and clinical outcomes of multidrug-resistant, gram-negative bloodstream infections in a European tertiary pediatric hospital during a 12-month period. Pediatr Infect Dis J 33: 929–932. 10.1097/INF.0000000000000339 24642515

[6] Zhang Q, Smith JC, Zhu Q, Guo Z, MacDonald NE (2012) A five-year review of Pseudomonas aeruginosa bacteremia in children hospitalized at a single center in southern China. Int J Infect Dis 16: e628–632. 10.1016/j.ijid.2012.03.014 22709682

[7] Peng Y, Bi J, Shi J, Li Y, Ye X, Chen X, et al (2014) Multidrug-resistant Pseudomonas aeruginosa infections pose growing threat to health care-associated infection control in the hospitals of Southern China: a case-control surveillance study. Am J Infect Control 42: 1308–1311. 10.1016/j.ajic.2014.08.006 25444305

[8] Ciofi Degli Atti M, Bernaschi P, Carletti M, Luzzi I, Garcia-Fernandez A, Bertaina A, et al (2014) An outbreak of extremely drug-resistant Pseudomonas aeruginosa in a tertiary care pediatric hospital in Italy. BMC Infect Dis 14: 494 10.1186/1471-2334-14-494 25209325 PMC4167521

[9] Langton Hewer SC, Smyth AR (2014) Antibiotic strategies for eradicating Pseudomonas aeruginosa in people with cystic fibrosis. Cochrane Database Syst Rev 11: CD004197 10.1002/14651858.CD004197.pub4 25383937

[10] Joo EJ, Kang CI, Ha YE, Kang SJ, Park SY, Chung DR, et al (2011) Risk factors for mortality in patients with Pseudomonas aeruginosa bacteremia: clinical impact of antimicrobial resistance on outcome. Microb Drug Resist 17: 305–312. 10.1089/mdr.2010.0170 21381966

[11] Lewis GJ, Fang X, Gooch M, Cook PP (2012) Decreased resistance of Pseudomonas aeruginosa with restriction of ciprofloxacin in a large teaching hospital's intensive care and intermediate care units. Infect Control Hosp Epidemiol 33: 368–373. 10.1086/664763 22418632

[12] Tumbarello M, De Pascale G, Trecarichi EM, Spanu T, Antonicelli F, Maviglia R, et al (2013) Clinical outcomes of Pseudomonas aeruginosa pneumonia in intensive care unit patients. Intensive Care Med 39: 682–692. 10.1007/s00134-013-2828-9 23370828

[13] Micek ST, Wunderink RG, Kollef MH, Chen C, Rello J, Chastre J, et al (2015) An international multicenter retrospective study of Pseudomonas aeruginosa nosocomial pneumonia: Impact of multidrug resistance. Crit Care 19: 219 10.1186/s13054-015-0926-5 25944081 PMC4446947

[14] Sax H, Allegranzi B, Chraiti MN, Boyce J, Larson E, Pittet D. (2009) The World Health Organization hand hygiene observation method. Am J Infect Control 37: 827–834. 10.1016/j.ajic.2009.07.003 20004812

[15] O'Grady NP, Alexander M, Burns LA, Dellinger EP, Garland J, Heard SO, et al (2011) Guidelines for the prevention of intravascular catheter-related infections. Am J Infect Control 39: S1–34. 10.1016/j.ajic.2011.01.003 21511081

[16] Haley RW, Culver DH, White JW, Morgan WM, Emori TG, Munn VP, et al (1985) The efficacy of infection surveillance and control programs in preventing nosocomial infections in US hospitals. Am J Epidemiol 121: 182–205. 4014115 10.1093/oxfordjournals.aje.a113990

[17] Niederman MS (2010) Hospital-acquired pneumonia, health care-associated pneumonia, ventilator-associated pneumonia, and ventilator-associated tracheobronchitis: definitions and challenges in trial design. Clin Infect Dis 51 Suppl 1: S12–17. 10.1086/653035 20597660

[18] Institute CaLS Performance standards for antimicrobial susceptibility testing; (CLSI) tis, editor: Wayne. M100-S118 p.

[19] Rex JH, Pfaller MA, Lancaster M, Odds FC, Bolmstrom A, Rinaldi MG. (1996) Quality control guidelines for National Committee for Clinical Laboratory Standards—recommended broth macrodilution testing of ketoconazole and itraconazole. J Clin Microbiol 34: 816–817. 8815089 10.1128/jcm.34.4.816-817.1996 PMC228898

[20] Tumbarello M, Repetto E, Trecarichi EM, Bernardini C, De Pascale G, Parisini A, et al (2011) Multidrug-resistant Pseudomonas aeruginosa bloodstream infections: risk factors and mortality. Epidemiol Infect 139: 1740–1749. 10.1017/S0950268810003055 21226988

[21] Proulx F, Gauthier M, Nadeau D, Lacroix J, Farrell CA (1994) Timing and predictors of death in pediatric patients with multiple organ system failure. Crit Care Med 22: 1025–1031. 8205810 10.1097/00003246-199406000-00023

[22] Parker CM, Kutsogiannis J, Muscedere J, Cook D, Dodek P, Day AG, et al (2008) Ventilator-associated pneumonia caused by multidrug-resistant organisms or Pseudomonas aeruginosa: prevalence, incidence, risk factors, and outcomes. J Crit Care 23: 18–26. 10.1016/j.jcrc.2008.02.001 18359417

[23] Anthony M, Bedford-Russell A, Cooper T, Fry C, Heath PT, Kennea N, et al (2013) Managing and preventing outbreaks of Gram-negative infections in UK neonatal units. Arch Dis Child Fetal Neonatal Ed 98: F549–553. 10.1136/archdischild-2012-303540 23792354

[24] Soule BM, Huskins WC (1997) A global perspective on the past, present, and future of nosocomial infection prevention and control. Am J Infect Control 25: 289–293. 9276539 10.1016/s0196-6553(97)90019-5

[25] Oncul O, Oksuz S, Acar A, Ulkur E, Turhan V, Uygur F, et al (2014) Nosocomial infection characteristics in a burn intensive care unit: analysis of an eleven-year active surveillance. Burns 40: 835–841. 10.1016/j.burns.2013.11.003 24296064

[26] Kaminski C, Timsit JF, Dubois Y, Zahar JR, Garrouste-Orgeas M, Vesin A, et al (2011) Impact of ureido/carboxypenicillin resistance on the prognosis of ventilator-associated pneumonia due to Pseudomonas aeruginosa. Crit Care 15: R112 10.1186/cc10136 21481266 PMC3219393

[27] Farias JA, Frutos F, Esteban A, Flores JC, Retta A, Baltodano A, et al (2004) What is the daily practice of mechanical ventilation in pediatric intensive care units? A multicenter study. Intensive Care Med 30: 918–925. 15029473 10.1007/s00134-004-2225-5 PMC7095496

[28] Lopes JM, Goulart EM, Starling CE (2007) Pediatric mortality due to nosocomial infection: a critical approach. Braz J Infect Dis 11: 515–519. 17962879 10.1590/s1413-86702007000500013

[29] Chen XX, Lo YC, Su LH, Chang CL (2014) Investigation of the case numbers of catheter-related bloodstream infection overestimated by the central line-associated bloodstream infection surveillance definition. J Microbiol Immunol Infect pii: S1684–1182(14). 10.1016/j.jmii.2014.03.006 24856425

[30] Rajkumari N, John NV, Mathur P, Misra MC (2014) Antimicrobial Resistance in Pseudomonas sp. Causing Infections in Trauma Patients: A 6 Year Experience from a South Asian Country. J Glob Infect Dis 6: 182–185. 10.4103/0974-777X.145250 25538457 PMC4265834

[31] Veltman ES, Vos FJ, Meis JF, Goosen JH (2015) Debridement, antibiotics and implant retention in early postoperative infection with Pseudomonas aeruginosa. J Infect 70:307–309. 10.1016/j.jinf.2014.10.002 25445886

[32] Saini H, Chhibber S, Harjai K (2015) Azithromycin and ciprofloxacin: A possible synergistic combination against Pseudomonas aeruginosa biofilm-associated urinary tract infections. Int J Antimicrob Agents 45:359–367. 10.1016/j.ijantimicag.2014.11.008 25604277

[33] Touw DS, Patel DR, van den Berg B (2010) The crystal structure of OprG from Pseudomonas aeruginosa, a potential channel for transport of hydrophobic molecules across the outer membrane. PLoS One 5: e15016 10.1371/journal.pone.0015016 21124774 PMC2993939

[34] Giske CG, Monnet DL, Cars O, Carmeli Y, ReAct-Action on Antibiotic R (2008) Clinical and economic impact of common multidrug-resistant gram-negative bacilli. Antimicrob Agents Chemother 52: 813–821. 18070961 10.1128/AAC.01169-07 PMC2258516

[35] Di Pasquale M, Ferrer M, Esperatti M, Crisafulli E, Giunta V, Li Bassi G, et al (2014) Assessment of severity of ICU-acquired pneumonia and association with etiology. Crit Care Med 42: 303–312. 10.1097/CCM.0b013e3182a272a2 23989176

[36] Joseph NM, Devi S, Shashikala P, Kanungo R (2013) Changing Trend in the Antibiotic Resistance Pattern of Pseudomonas Aeruginosa Isolated from Wound Swabs of Out-Patients and in-Patients of a Tertiary Care Hospital. J Clin Diagn Res 7: 2170–2172. 10.7860/JCDR/2013/6113.3461 24298467 PMC3843476

